# New Insights Into Drug Discovery Targeting Tau Protein

**DOI:** 10.3389/fnmol.2020.590896

**Published:** 2020-12-03

**Authors:** Yoshiyuki Soeda, Akihiko Takashima

**Affiliations:** Laboratory for Alzheimer's Disease, Department of Life Science, Faculty of Science, Gakushuin University, Tokyo, Japan

**Keywords:** tau protein, post-translational modifications, aggregation, microtubule stabilizer, immunotherapy, oligonucleotide therapy, liquid-liquid phase separation, inflammation

## Abstract

Microtubule-associated protein tau is characterized by the fact that it is an intrinsically disordered protein due to its lack of a stable conformation and high flexibility. Intracellular inclusions of fibrillar forms of tau with a β-sheet structure accumulate in the brain of patients with Alzheimer's disease and other tauopathies. Accordingly, detachment of tau from microtubules and transition of tau from a disordered state to an abnormally aggregated state are essential events preceding the onset of tau-related diseases. Many reports have shown that this transition is caused by post-translational modifications, including hyperphosphorylation and acetylation. The misfolded tau is self-assembled and forms a tau oligomer before the appearance of tau inclusions. Animal and pathological studies using human samples have demonstrated that tau oligomer formation contributes to neuronal loss. During the progression of tauopathies, tau seeds are released from cells and incorporated into other cells, leading to the propagation of pathological tau aggregation. Accumulating evidence suggests several potential approaches for blocking tau-mediated toxicity: (1) direct inhibition of pathological tau aggregation and (2) inhibition of tau post-translational modifications that occur prior to pathological tau aggregation, (3) inhibition of tau propagation and (4) stabilization of microtubules. In addition to traditional low-molecular-weight compounds, newer drug discovery approaches such as the development of medium-molecular-weight drugs (peptide- or oligonucleotide-based drugs) and high-molecular-weight drugs (antibody-based drugs) provide alternative pathways to preventing the formation of abnormal tau. Of particular interest are recent studies suggesting that tau droplet formation by liquid-liquid phase separation may be the initial step in aberrant tau aggregation, as well results that implicate roles for tau in dendritic and nuclear functions. Here, we review the mechanisms through which drugs can target tau and consider recent clinical trials for the treatment of tauopathies. In addition, we discuss the utility of these newer strategies and propose future directions for research on tau-targeted therapeutics.

## Introduction

**Figure 1:**
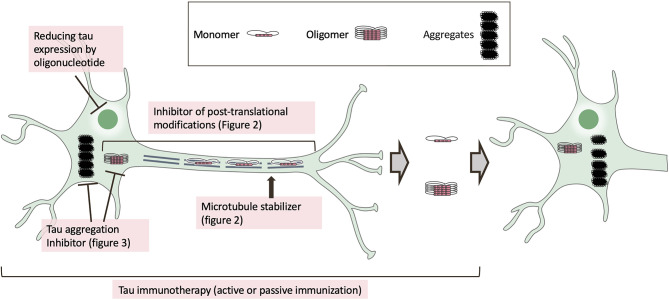
Mechanism of tau-targeted drugs in clinical trials. In tauopathies, tau protein is dissociated from microtubules by post-translational modifications, including phosphorylation, and tau is mis-sorted into the somatodendritic compartment. The mis-sorted tau undergoes further post-translational modifications and is converted to misfolded tau. After tau self-assembly, tau filaments are formed via tau oligomers. The pathological tau seed is subsequently released from the pre-synapse and propagated into post-synapses. Tau-based drugs in clinical trials are inhibitors of post-translational modification (Figure 2) or tau aggregation inhibitors (Figure 3), as well as oligonucleotides to reduce tau expression, microtubule stabilizers, and immunotherapeutics.

Two classes of drugs for dementia treatment have been approved by the major regulatory agencies (US Food and Drug Administration, FDA; European Medicines Agency, EMA; Pharmaceuticals and Medical Devices Agency, PMDA): acetylcholinesterase inhibitors, which treat mild to moderate Alzheimer's disease (AD), and N-methyl-D-aspartate receptor antagonists (e.g., memantine), which treat moderate to severe AD. Although these drugs can slow progression and control dementia-related symptoms, the treatments are not definitive. Systematic reviews have reported that the drugs are efficacious against dementia (Loveman et al., 2006; Tan et al., 2014), but they are also controversial in terms of cost effectiveness (Bond et al., 2012). There are currently many research and development efforts to provide disease-modifying therapies for AD treatment (Cummings et al., 2019). The main targets are amyloid-β (Aβ), a major component of senile plaques, and tau, neurofibrillary tangles (NFT). To investigate the Aβ cascade hypothesis, many clinical trials of therapeutic approaches targeting Aβ have been conducted. However, clinical trials for targeting Aβ have repeatedly failed (Holmes et al., 2008; Rosenblum, 2014). Because histological analysis and tau positron emission tomographic studies have revealed that cognitive impairment correlate better with tau and neuronal loss than with Aβ pathology (Bondareff et al., 1989; Bobinski et al., 1996; Gomez-Isla et al., 1996; Guillozet et al., 2003; Schöll et al., 2016; Schwarz et al., 2016; Bejanin et al., 2017), AD drug discovery research has increasingly shifted from Aβ to tau protein (Giacobini and Gold, 2013), with some reaching clinical trial stages. In this review, we describe and discuss the structure and mechanisms of action of drugs that target tau (Figure 1). We also discuss perspectives for drug development in this area.

## Tau Protein and Tauopathies

Tau is a stabilizing microtubule-associated protein that was discovered in 1975 (Weingarten et al., 1975). The protein is mainly found in the axonal compartment of neurons (Morris et al., 2011; Mandelkow and Mandelkow, 2012; Guo et al., 2017). In the adult human brain, alternative splicing from the *MAPT* gene on chromosome 17 yields six tau isoforms (352–441 amino acid residues; 37–46 kDa) (Goedert et al., 1991), which are distinguished by the absence or presence of one or two N-terminal inserts, and the presence of three (3R) or four (4R) microtubule-binding repeats in the C-terminal half of tau (Guo et al., 2017).

Tauopathies are neurological disorders (Avila et al., 2004; Götz and Götz, 2019), characterized by aberrant tau aggregates (NFT and tau inclusions) in neurons and glial cells. These aggregates represent tau gene mutations or hyperphosphorylated tau (Kovacs, 2015). The majority of tauopathic patients also show depositions of Aβ, α-synuclein, or huntingtin (Guo et al., 2017). These observations suggest that tau abnormalities have a common pathological role across neurodegenerative diseases.

AD is the most common and best-studied tauopathy. The disease is caused by extensive atrophy of the brain beginning in the temporal and parietal lobes. Analysis of cell lysates from the AD brain by sodium dodecyl sulfate polyacrylamide gel electrophoresis reveals three major electrophoresis bands: tau proteins with relative molecular weights of 68,000, 64,000, and 60,000 (Lee et al., 1991; Goedert et al., 1992; Greenberg et al., 1992; Delacourte et al., 1999). Although the actual molecular weight of tau is 37–46 kDa, treatment of AD-derived samples with phosphatases shows that the band pattern of tau was similar to that of recombinant human tau (Hanger et al., 2002). This finding indicates that the tau aggregates found in AD undergo post-translational modifications (Guo et al., 2017). Indeed, structural biology studies have revealed that the major components of tangles in AD are paired helical filaments (PHF) and straight filaments (SF), and both types are composed primarily of abnormally phosphorylated tau proteins (Kosik et al., 1988; Gendron and Petrucelli, 2009). Like the tangles in the healthy adult human brain, those in the AD brain consist of 3R and 4R isoforms (1:1 ratio) (Williams, 2006). However, some other tauopathies are characterized by an imbalance in the ratio of 4R/3R tau isoforms. For example, brains from patients with progressive supranuclear palsy (PSP) and corticobasal degeneration (CBD) predominantly exhibit 4R tau, whereas the insoluble tau of Pick's disease (PiD) is mainly 3R tau (Arai et al., 2003). In frontotemporal dementia and parkinsonism linked to chromosome 17 (FTDP-17), the predominance of isoforms varies according to the type of disease-causing tau mutation (de Silva et al., 2006; Andreadis, 2012; Rossi and Tagliavini, 2015).

## Tau-Targeted Therapies

Tau-targeted drugs may be a promising disease-modifying therapy because previous studies focusing on the correlation of AD neuropathological changes (Aβ plaques and NFT) with cognitive impairment have shown that the severity of cognitive impairment correlated best with the burden of abnormal tau (Nelson et al., 2012). Accordingly, many clinical trials of drugs targeting tau have been conducted.

### Post-translational Modifications

**Figure 2:**
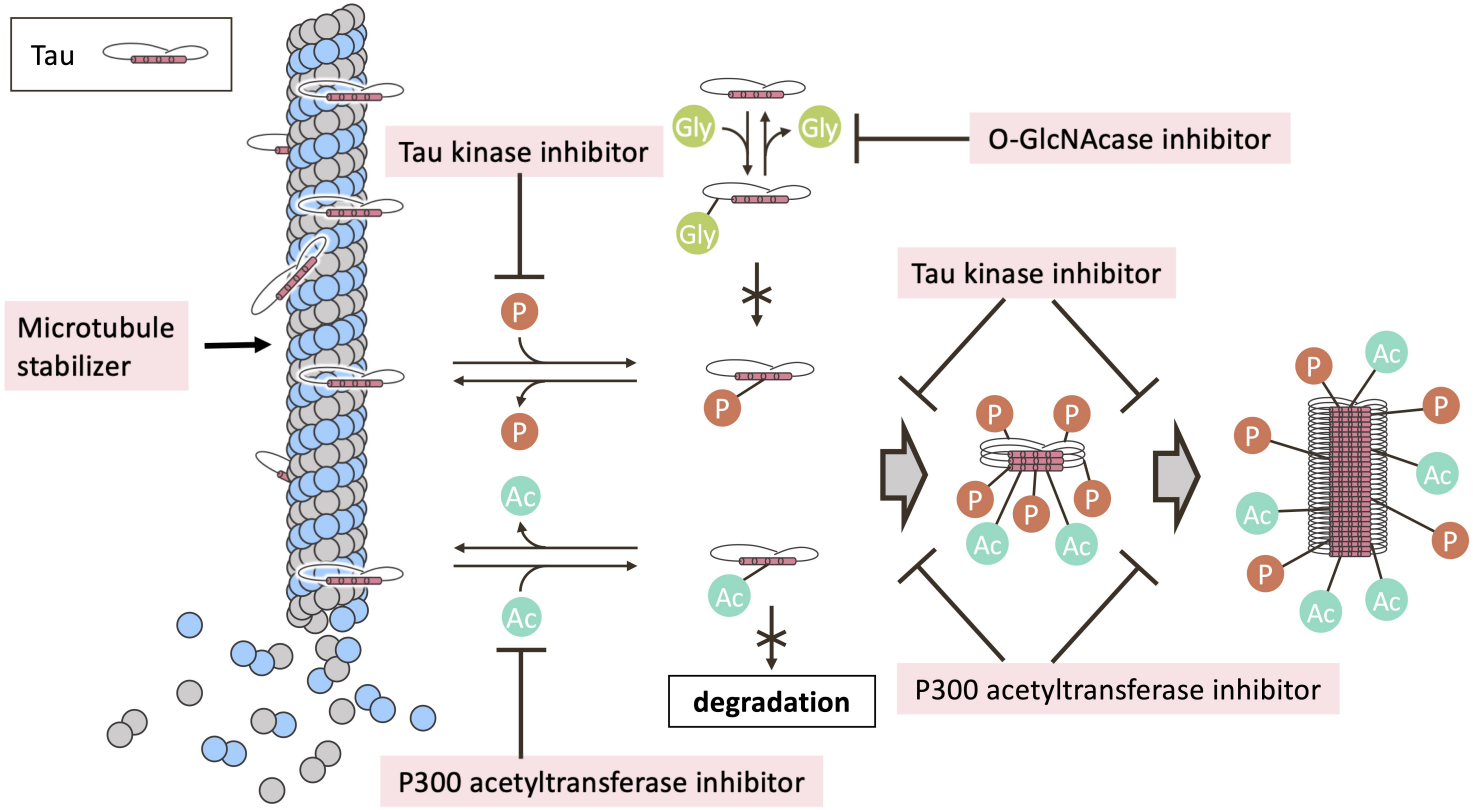
Mechanism of tau post-translational modification inhibitors in clinical trials. Post-translational modifications, including phosphorylation and acetylation, regulate the binding of tau to microtubules. Microtubule instability and depolymerization are observed in tauopathies, suggesting a therapeutic role for microtubule stabilizers. Phosphorylation, acetylation, or both, enhance tau aggregation. O-GlcNAcylation at serine and threonine compete with phosphorylation of the same residues. Tau degradation is inhibited by acetylation. The post-translational modifications are tightly regulated by various enzymes that mediate the addition and removal of the modifying groups. In clinical trials, tau kinase inhibitors or P300 acetyltransferase inhibitors have been investigated for their ability to inhibit tau phosphorylation or tau acetylation. The usefulness of O-GlcNAcase inhibitors to elevate tau O-GlcNAcylation has also been examined in clinical trials. Ac, acetylation; Gly, O-GlcNAcylation; P, phosphorylation.

Tau undergoes a variety of post-translational modifications, including phosphorylation, acetylation, glycation, nitration, addition of β-linked N-acetylglucosamine (O-GlcNAcylation), oxidation, polyamination, sumoylation, and ubiquitination (Martin et al., 2011; Morris et al., 2015). Here, we discuss some of the post-translational modifications of tau, its function and relationship to disease, and drugs that have been developed to prevent or ameliorate these modifications (Figure 2).

#### Tau Phosphorylation

Phosphorylation is the best known post-translational modification of tau. Tau bears 85 phosphorylation sites, including 45 serine residues, 35 threonine residues, and five tyrosine residues (Hanger et al., 2009). Tau phosphorylation regulate its binding to microtubules (Gong and Iqbal, 2008). Phosphorylation of tau, including at residues S262, S293, S324, and S356, cause a dissociation of the bond between tau and microtubules, whereas these bonds are enhanced when tau is hypophosphorylated (Mandelkow et al., 1995; Martin et al., 2011). NFT in the AD brain are accumulations of PHF and SF comprised of hyperphosphorylated tau (Gendron and Petrucelli, 2009). Under normal conditions, there is an average 2–3 moles of phosphate per molecule of tau is present, but in AD, this ratio can be about 3–4 times higher (Gong and Iqbal, 2008). Tau hyperphosphorylation induces accumulation of tau in the somatodendritic compartment (mis-sorting), self-aggregation, polymerization, defects of axonal mitochondrial trafficking, ultimately leading to neuronal toxicity (Zempel et al., 2010; Zempel and Mandelkow, 2019; Lauretti and Praticò, 2020). Tau phosphorylation precedes the formation of tau fibrils in the AD brain (Iqbal et al., 2005). Together, these observations suggest that tau hyperphosphorylation is involved in the development and pathogenesis of tauopathies and that its inhibition may be a therapeutic strategy.

Tau phosphorylation is tightly controlled by the balance of protein kinase and phosphatase activity (Hanger et al., 2009). Tau is a major substrate of protein phosphatase 2A (PP2A) whose activity is reduced in the AD brain (Gong et al., 1993, 1995; Sontag et al., 2004; Liu et al., 2005). However, because of its substrate specificity and several regulatory subunits, PP2A is not easily amenable to drug targeting (Wolfe, 2016). Therefore, attention has been paid to developing protein kinase inhibitors that can reduce tau aggregation and neuronal death in tauopathies (Hanger et al., 2009).

##### Glycogen Synthase Kinase 3β

Of the tau amino acid residues observed to be phosphorylated in AD, at least 26 sites have been identified as targets of glycogen synthase kinase 3 (GSK3). Indeed, the total protein level and activity of GSK3 in brains with tauopathies seem to correlate with the progression of neurodegeneration (Yamaguchi et al., 1996; Imahori and Uchida, 1997; Pei et al., 1997), and over-activation of GSK3β contributes to tau hyperphosphorylation (Blalock et al., 2004; Guo et al., 2017). Interestingly, Aβ activates GSK3β and tau hyperphosphorylation and, subsequently, neuronal death (Takashima et al., 1993, 1996; Takashima, 2006). While non-phosphorylated recombinant tau is polymerized in the presence of an inducer of arachidonic acid, the phosphorylation of tau by GSK3β promotes polymerization (Rankin et al., 2007). Other supporting data includes demonstration that co-transfection of truncated tau at D421 and GSK3β (Cho and Johnson, 2004), or triple expression of wild-type tau, GSK3β and JNK (Sato et al., 2002) in cultured cells leads to the formation of detergent-insoluble tau and thioflavin-S-positive inclusions. In transgenic mice, lithium or NP12, a pharmacological inhibitor of GSK3β, causes a reduction of tau phosphorylation and NFT formation (Caccamo et al., 2007; Leroy et al., 2010) and also restored the loss of neurons (Serenó et al., 2009). Based on this knowledge, an open-label trial of lithium for PSP syndrome and CBD (www.ClinicalTrials.gov Identifier: NCT00703677), but was discontinued because of poor tolerability (Panza et al., 2020). The TAUROS trial to evaluate another GSK3β inhibitors (tideglusib) showed that while the test drug reduced brain atrophy in PSP patients (Höglinger et al., 2014) it failed to demonstrate clinical efficacy in patients with mild to moderate PSP (Tolosa et al., 2014).

##### Cyclin-Dependent Kinase 5

At least 17 kinases have been identified as tau phosphorylation kinases (Martin et al., 2013), with GSK3β and cyclin-dependent kinase 5 (CDK5) being the most frequently reported among them. CDK5 is a proline-directed serine/threonine-protein kinase. Physiological activation is controlled by binding the regulatory subunit, p35 or p39, to CDK5, leading to brain development and synaptic activity. The p35 and p39 are cleaved by calpain, producing p25 or p29. The binding of p25 or p29 to CDK5 leads to pathological hyperactivation (Kimura et al., 2014a). CDK5 phosphorylates tau at 9–13 sites (Kimura et al., 2014a). CDK5 was also found in the neurons having pretangle or NFT (Pei et al., 1998). An experiment on cross-transgenic mice overexpressing p25 and P301L tau transgenic mice (JNPL3) showed increased tau phosphorylation level and number of NFT (Noble et al., 2003). The silencing of CDK5 by si-RNA reduced tau phosphorylation in triple-transgenic AD mice (Piedrahita et al., 2010). Roscovitine is a small-molecule drug that inhibits CDK5 activity. CDK5 is also involved in various cancers (Pozo and Bibb, 2016); therefore, clinical trials on roscovitine have been conducted in patients with cancer (Cicenas et al., 2015). No trials on CDK5 inhibitors have been reported in tauopathies. Notably, Wen et al. reported that administration of CP681301, a CDK5 inhibitor, enhanced tau phosphorylation in p25 overexpression transgenic mice (Wen et al., 2008). CDK5 indirectly phosphorylates GSK3β at S9 and inhibits its activity (Engmann and Giese, 2009), suggesting that CDK5 inhibition enhances tau phosphorylation by activating GSK3β. As CDK5 can phosphorylate molecules other than tau, therapeutic agents targeting CDK5 should be developed with great caution.

##### Fyn

Tau protein has five tyrosine residues (18, 29, 197, 310, and 394 sites) that are phosphorylated by tyrosine kinases. Src family kinase, including Fyn, modulates neurotransmitter function and NMDA trafficking (Ohnishi et al., 2011). Interestingly, tau reduction improved Aβ-induced cognitive impairments in J20 transgenic mice that express a human APP with the Swedish (K670M/N671L) and Indiana (V717F) mutants (Roberson et al., 2007; Yoshikawa et al., 2018). Fyn is located at the PSD95-rich post-synapse by binding to tau and phosphorylates the NMDA receptor subunit NR2b. This complex promoted Aβ-induced excitotoxicity (Ittner et al., 2010). Fyn preferentially phosphorylated Tyr18 among the five tyrosine residues in tau (Scales et al., 2011). Biochemical and immunocytochemical assays showed that phosphorylated tau at Y18 was observed in the NFT from the AD brain (Lee et al., 2004). Fyn deficiency reduced tau NFT formation and hyperphosphorylation in mice overexpressing P301L-tau (Liu et al., 2020). These facts suggest that Fyn inhibition is a potential target for tauopathy treatment. Saracatinib is a small-molecule inhibitor of Fyn. Preclinical studies showed that saracatinib rescued synaptic depletion and spatial memory deficits in APP (Swe)/presenilin 1(ΔE9) mice (Kaufman et al., 2015; Smith et al., 2018). A phase 1b trial in mild and moderate AD showed that saracatinib is safe, has good tolerability, and can penetrate into the central nervous system (Nygaard et al., 2015). Unfortunately, the phase 2 trial showed no positive therapeutic effects of the drug in patients with AD (van Dyck et al., 2019).

##### Thousand-and-One Amino Acid Kinases

Recently, thousand-and-one amino acid kinases (TAOKs) have been identified as tau kinases, which may be involved in the onset of AD pathology and dementia (Tavares et al., 2013; Giacomini et al., 2018). TAOKs are referred to as prostate-derived sterile 20-like kinases (PSKs), i.e., serine/threonine kinase. TAOKs have two isoforms: TAOK1 (PSK2) and TAOK2 (PSK1). TAOKs phosphorylated ≥40 sites on recombinant human tau (Tavares et al., 2013). High TAOK activation (pS181) was observed in NFT and pretangles of the entorhinal cortex in subjects with Braak stage II but not in control subjects (Giacomini et al., 2018). A TAOK inhibitor, compound 43, inhibited tau phosphorylation at AT8 and 12E8 sites *in vitro* and *in vivo* (Giacomini et al., 2018). Furthermore, the drug also inhibited phosphorylation at T123 and T427 sites, newly found in AD (Giacomini et al., 2018), suggesting that TAOKs may be a novel target to improve tau-related pathogenesis. A previous report showed that TAOKs modulate microtubule dynamics and organization (Mitsopoulos et al., 2003). Compound 43 promoted cell death in a cultured cancer cell line (Koo et al., 2017). These findings suggest that the development of TAOK inhibitor should proceed with caution.

Because many tau kinases are involved in physiological intracellular signaling pathways, tau kinase inhibitor development appropriately avoiding physiological on targets might be difficult. Meanwhile, based on the view that a specific phosphorylation pattern is required to induce tau self-assembly (Fichou et al., 2019; Lauretti and Praticò, 2020), several groups have reported data indicating that the phospho-S396/404 epitope constitutes an effective therapeutic target (Boutajangout et al., 2011; Gu et al., 2013; Liu et al., 2016; Rosenqvist et al., 2018). Thus, studies have used immunotherapy targeting tau phosphorylation at S396/404. In a preclinical study, subcutaneous injection of the liposome-based vaccine ACI-35 into wild-type mice and mice carrying the P301L tau mutation induced the formation of antibodies specific to phospho-S396 and S404 tau and reduced soluble and insoluble tau in the brain. This vaccine also improved body weight loss and clasping frequency and survival (Theunis et al., 2013). Thus, far, ACI-35 has been used in a phase 1 trial (Main ID in the WHO International Clinical Trials Registry Platform: ISRCTN13033912) (see section on *Tau Clearance and Immunotherapy*).

#### Tau Acetylation

There are ≥30 lysine residues that are potentially acetylated in the tau sequence (Kontaxi et al., 2017), mainly located in the proline-rich region, the microtubule-binding region, and the C-terminal domain (Kontaxi et al., 2017). The level of their acetylation is regulated by acetyltransferases (p300 and CREB-binding protein; Min et al., 2010; Cohen et al., 2011; Cook et al., 2014b) and deacetylases (histone deacetylase 6 and sirtuin 1; Cook et al., 2014a). Tau proteins promote the self-acetylation of autologous lysine residues via catalytic cysteine residues (C291 and C322) in the microtubule-binding domain (Cohen et al., 2013). Lysine residues in tau are more highly acetylated in the brains of AD and other tauopathy patients than in healthy brains (Irwin et al., 2012, 2013). Specific acetylation at residues K280/K281 on tau inhibits microtubule stabilization and promotes fibrillar tau aggregate formation (Trzeciakiewicz et al., 2017). An increase in acetylated tau by deletion of sirtuin 1, a class III protein deacetylase, inhibits its degradation, leading to the accumulation of pathogenic phospho-tau *in vivo* (Min et al., 2010). These facts suggest that tau acetylation may be important for tau-induced toxicity.

Salsalate is an old salicylate derivative which has with anti-inflammatory properties related to its ability to inhibit activation of the NF-κB pathway (Panza et al., 2019). Min et al. reported that salsalate inhibits tau acetylation by blocking p300 acetyltransferase activity and acetylation of K174 in the PS19 transgenic mouse line which overexpresses P301S-tau. Moreover, these authors found that salsalate prevents hippocampal atrophy and memory impairment (Min et al., 2015). An open-label pilot study (phase 1) of salsalate (2,250 mg/day) in 10 PSP patients found that although salsalate was safe and well-tolerated, the drug did not significantly improve cognitive performance in patients (VandeVrede et al., 2020b). This may be explained by either the poor penetration of salsalate into the brain (<3%), or by an increase in tau aggregation following reduced tau acetylation.

#### Tau Ubiquitination

Lysine residues undergo not only acetylation but also ubiquitination which is closely related to the proteasomal degradation pathway (Cook et al., 2014b). Hyperphosphorylated tau is ubiquitinated in patients with AD (Mori et al., 1987; Perry et al., 1987; Cripps et al., 2006) and interestingly, dysfunction of either the proteasomal or lysosomal degradation pathways may lead to accumulation of excessive ubiquitinated tau species in AD patients that can contribute to NFT formation in disease (Wang and Mandelkow, 2012; Cook et al., 2014b). Given this, it is plausible that tau acetylation competes with ubiquitination and therefore reduces tau ubiquitination and NFT formation. Another observation warranting the role of tau acetylation in tauopathies is thar Aβ-induced tau bead (mostly acetylated and oligomeric tau) formation in neurites is inhibited by the HDAC6 inhibitor, Tubastatin A (Tseng et al., 2017).

#### O-GlcNAcylation

Glycosylated tau is present in PHF from Alzheimer disease brains (Wang et al., 1996). The addition of β-linked N-acetylglucosamine (O-GlcNAcylation) is the non-canonical form is glycosylation, and the levels are strictly regulated by O-GlcNAc transferase and neutral β-hexosaminidase known as O-GlcNAcase (OGA). Since serine and threonine residues undergo O-GlcNAcylation (Arnold et al., 1996), there is a competition between O-GlcNAcylation and phosphorylation (Liu et al., 2004; Hart et al., 2007; Di Domenico et al., 2019). In P301L tau transgenic mice (JNPL3), an OGA inhibitor was found to increase tau O-GlcNAcylation, thereby inhibiting the formation of tau aggregates and neuronal loss (Yuzwa et al., 2012). In the AD brain, reduction of tau O-GlcNAcylation (Liu et al., 2004; Wang et al., 2016) is linked to neurofibrillary pathology (Liu et al., 2009). On the other hand, forebrain-specific O-GlcNAc transferase conditional knockout mice display increased neurodegeneration and tau phosphorylation and cognitive impairment (Wang et al., 2016). These findings suggest that upregulation of tau O-GlcNAcylation may be a therapeutic strategy for tau-related neurodegeneration.

Thiamet G is an inhibitor of OGA, reportedly with good bioavailability (Yu et al., 2012; Yuzwa et al., 2012; Borghgraef et al., 2013). Acute injection of thiamet G into the lateral ventricle of wild-type tau transgenic mice decreased the site-specific phosphorylation of T181, T212, S214, S262/S356, S404, and S409 residues (Yu et al., 2012). Also, oral administration of thiamet G in the drinking water increased O-GlcNAcylation, and inhibited tau aggregates and neuronal cell loss (Yuzwa et al., 2012). A low-molecular-weight OGA inhibitor, MK-8719, developed in a collaboration between Alectos Therapeutics and Merck, was found to elevate brain O-GlcNAc levels, reduce pathological tau, and ameliorate brain atrophy in an rTg4510 mouse model of tauopathy (Wang et al., 2020). Recently, a clinical trial in 16 healthy controls showed that MK-8719 was well-tolerated (VandeVrede et al., 2020a). Administration of another OGA inhibitor, ASN120290 (developed by Asceneuron) to P301S transgenic mice leads to increased O-GlcNAcylated tau and decreased tau phosphorylation (VandeVrede et al., 2020a); subsequently ASN120290 was found to be safe and well-tolerated in a phase 1 study involving 61 healthy volunteers (VandeVrede et al., 2020a).

## Tau Aggregation

Onset and progression of tauopathies involve the formation of misfolded and oligomerized tau and the appearance of NFT. Classically, NFT gradually overload nerve cells and eventually cause neuronal cell death (Ward et al., 2012; Guo et al., 2017). The appearance of tau deposition is a typical pathological sign in many tauopathies, including AD, and has been used to classify disease stage in the Braak system (Braak and Braak, 1995). Tau is self-assembled through the microtubule-binding domain and then converted to aggregates. In the microtubule-binding region, at least two amino acid sequences are involved in tau aggregation (Schweers et al., 1995; von Bergen et al., 2000, 2001). Hexapeptide segments known as PHF6 (^306^VQIVYK^311^) and PHF6^*^ (^275^VQIINK^280^) are present in R3 and R2, respectively. The segments are enriched in hydrophobic amino acids, and inter-molecular interaction is essential for forming the β-sheet structure (von Bergen et al., 2000, 2001). *In vitro* and *in silico* experiments showed that intact tau monomer has a β-hairpin structure in regions including the PHF6 segment. In the presence of P301L-tau mutation, PHF6 was shifted to disfavor the local compact structure, which enhanced the aggregation propensity (Chen et al., 2019). Disulfide bridges formed between cysteine residues contribute to protein structure or protein–protein (peptide) interaction. Although the role of cysteine residues in tau aggregation remains disputable, some reports showed that an intermolecular disulfide bond is involved in the seed formation to initiate tau polymerization (Bhattacharya et al., 2001) and tau oligomer (Schweers et al., 1995; Sahara et al., 2007). Our finding that inhibition of tau oligomer formation by capping cysteine residues with 1,2-dihydroxybenzene provides support for the role of cysteine in the oligomer formation (Soeda et al., 2015). Hyper-phosphorylated tau at various sites is observed in NFT. It has been shown that tau kinase inhibitors reduce tau phosphorylation at multiple sites and inhibit tau aggregation (Lee et al., 2011; Noble et al., 2020). While tau phosphorylation at specific sites promotes tau aggregation (Jeganathan et al., 2008; Despres et al., 2017), phosphorylation at some sites inhibits tau aggregation (Schneider et al., 1999). The facts suggest that compounds that directly target tau aggregation may be more effective tauopathies than tau kinase inhibitors. Here, we describe tau aggregation inhibitors.

The recombinant tau protein is polymerized in the presence of polyanion, including heparin (Goedert et al., 1996) or RNA (Kampers et al., 1996), and the aggregation level can be monitored by fluorescent dye Thioflavin-T (S). Using this experimental system, many researchers screened tau aggregation inhibitors (Taniguchi et al., 2005; Bulic et al., 2009; Crowe et al., 2009). Many of the aggregation inhibitors discovered share a common characteristic: a negative or positive charge in their structure, antioxidant properties, and natural compounds.

### Curcumin

Curcumin is a primary component of the Indian turmeric spice extracted from the rhizome of *Curcuma longa*. Turmeric is an herbal medicine used to treat respiratory conditions, abdominal pain, sprains, and swelling (Chen et al., 2018), and curcumin has multifaceted actions as antioxidant, anti-angiogenic, anti-inflammatory, and neuroprotective effects (Maheshwari et al., 2006). Due to these actions, curcumin has been repeatedly reported to have potential benefit for cognitive function (Dong et al., 2012; Cox et al., 2015). Curcumin inhibits amyloidogenic protein aggregation, including not only Aβ (Ono et al., 2004) but also tau (Rane et al., 2017; Bijari et al., 2018). The inhibitory mechanisms of tau aggregation by curcumin are involved in the reduction of tau oligomer level (Rane et al., 2017) and the interaction to PHF6 segment (Bijari et al., 2018). These facts suggest that curcumin may contribute to tau-related neurodegeneration therapy. However, curcumin is poorly bioavailable and is rapidly degraded in the body (Vareed et al., 2008). Furthermore, clinical trials in AD showed no therapeutic benefit of curcumin (Chen et al., 2018). This has led to the development of analogs that improved bioavailability (Okuda et al., 2017; Lo Cascio et al., 2019). The results of clinical trials of these drugs are expected.

### Resveratrol

Resveratrol is a non-flavonoid polyphenol rich in grape skin and red wine (Xia et al., 2010). Resveratrol extends their lifespan in species, including mammals (Bauer et al., 2004; Viswanathan et al., 2005; Baur et al., 2006). Wine consumption has had beneficial effects on dementia (Orgogozo et al., 1997). These reports suggest that resveratrol may be beneficial for the treatment of AD. The Aβ fibrillary level was reduced by resveratrol in cultured cells (Feng et al., 2009; Ge et al., 2012) and APP/PS-1 transgenic mice (Porquet et al., 2014). Resveratrol inhibited the aggregation of the repeat domain of tau (K18) *in vitro* (PubChem BioAssay AID 1460, CID 445154). The level of tau phosphorylation at AT8 sites was reduced by resveratrol in P301L tau transgenic mice (JNPL3) (Yu et al., 2018). Resveratrol enhanced the tau dephosphorylation through PP2A activation (Schweiger et al., 2017) or downregulation of ERK1/2 and GSK3β signaling pathways (Jhang et al., 2017). The drug treatment rescued cognitive deficits in P301S tau transgenic mice (PS19) (Sun et al., 2019). Thus, resveratrol appears to both directly and indirectly inhibit tau aggregation. Alternatively, antioxidation and anti-inflammatory actions by resveratrol may contribute to the inhibitory effect on tau aggregation. Resveratrol has low bioavailability through rapid metabolism in the liver and intestine, leading to the development of nanocarriers and analogs (Chimento et al., 2019).

### Purpurin

Purpurin is a natural dye obtained from the madder extract and has an anthraquinone skeleton. *In vitro*, purpurin inhibited tau fibrillization by heparin through interaction with PHF6 segment (Viswanathan et al., 2020). Moreover, the drug broke down the pre-formed fibrils (Viswanathan et al., 2020). In Drosophila, overexpressing human tau, eye neurodegeneration was prevented by purpurin (Viswanathan et al., 2020). The purpurin permeability was observed in the cultured blood–brain barrier (BBB) model (Viswanathan et al., 2020), suggesting it may be suitable for the treatment of tau-related dementia.

### Ginseng

Ginseng is the root of Panax ginseng Meyer and has been used as an herbal medicine for various diseases. Red ginseng is believed to be a processed form of ginseng with enhanced pharmacological efficacy (Lee et al., 2015). Tau aggregation was inhibited by the red ginseng treatment *in vitro* (Shin et al., 2020b). As ginseng includes saponin and flavonoids (Choi, 2008), the inhibitory effect may be involved in the surfactant action or antioxidation by ginseng.

### Metal Nickel

The association between metals and neurodegenerative diseases, including AD, has been frequently reported (Aizenman and Mastroberardino, 2015). Focusing on the relationship between tau and metals, accumulation of iron (Spotorno et al., 2020) or aluminum (Walton, 2010) is associated with the NFT formation in patients with AD. Zinc (Huang et al., 2014), lead (Zhu et al., 2011), or aluminum (Shin et al., 1994) interacted with the microtubule-binding region or phosphorylation sites on tau, leading to aggregation *in vitro*. Metal nickel and its synthetic morpholine conjugate at 100 μM, conversely, inhibited tau aggregation *in vitro* (Gorantla et al., 2020). Unlike other metals, the inhibitory mechanism seems to be involved in the degradation and fragmentation of soluble tau. However, subcutaneous administration of nickel solution showed the accumulation of nickel and morphological changes in the liver, kidney, and spleen of mice (Pereira et al., 1998). Nickel concentrations in the liver, kidney, and spleen are 1.23–1.27 μg/g (9.79–10.11 nmol/g), 0.95–0.96 μg/g (7.56–7.64 nmol/g), and 4.96–4.98 μg/g (39.49–39.65 nmol/g), respectively (Pereira et al., 1998), indicating that the nickel administered to humans requires careful observation.

### Folic Acid

Low folate level in the serum is strongly associated with mild cognitive impairment (Quadri et al., 2004). Administration of folic acid (1.25 mg/day) improved cognitive scores for patients with AD treated with donepezil in a randomized trial (Chen et al., 2016), indicating that the supplementation may be clinically beneficial. An *in vitro* study showed that folic acid inhibited tau aggregation by stabilizing the tau native state (Ghasemzadeh and Riazi, 2020). Further, in cultured cells, folic acid reduced the tau phosphorylation level by regulating PP2A methylation (Li et al., 2015). Results of clinical trials on drugs against tauopathies besides AD are expected.

### Methylene Blue

Methylene blue (MB) or phenothiazine was first developed in the late 1800's for the treatment of malaria (VandeVrede et al., 2020a). MB was found *in vitro* to block tau–tau interaction and prevent self-aggregation (Wischik et al., 1996). Its administration to transgenic mice decreases the amount of phosphorylated tau aggregates (Hosokawa et al., 2012; Hochgrafe et al., 2015) and prevents memory impairment (Stack et al., 2014; Hochgrafe et al., 2015). Akuory et al. reported that MB inhibits tau aggregation by modifying cysteine residues in tau protein (Akoury et al., 2013) such that its disordered monomeric form is retained, preventing formation of filaments and their toxic precursors (Akoury et al., 2013). A phase 2 clinical trial of MB (Rember® TauRx, Singapore) in 321 patients with moderate AD showed that, as compared to placebo, 24 weeks of MB administration at a medium dose (138 mg/day) resulted in a significant improvement in AD subjects' ADAS-Cog score (Wischik et al., 2014). However, 200 mg of LMTM (LMTX, TRx0237), a MB-derived compound with greater tolerability and absorption, failed to achieve the pre-specified primary endpoint in two phase 3 trials involving ~1,700 patients with mild AD and 220 patients with the behavioral variant frontotemporal dementia (Gauthier et al., 2016; Wilcock et al., 2018). Because urine and stools were stained blue by MB and related compounds, blinding was deemed necessary; for this, a low dose of the active compound (8 mg/day) was given to the placebo group, but since a cohort analysis showed that the low dose might be effective (Wilcock et al., 2018), a phase-3 low-dose study of LMTM in AD (LUCIDITY; NCT03446001) was eventually launched.

A number of findings suggest that tau-induced toxicity is not due to tau filaments but rather to tau abnormality before the tau filaments (Kimura et al., 2010; Shafiei et al., 2017; Maeda and Takashima, 2019). For example, (i) the amount of neuronal loss in the AD brain exceeds the accumulation of NFT (Gómez-Isla et al., 1997) (ii) suppression of human tau in human P301L tau transgenic mice (rTg4510) by doxycycline does not inhibit tau filament formation but reduces neuronal loss (Santacruz et al., 2005); (iii) human Tau overexpression in *Drosophila*, results in neuronal loss without NFT formation (Wittmann et al., 2001); (iv) analysis of *in vivo* multiphoton imaging suggests that the formation of tangles is off-pathway to acute neuronal death (de Calignon et al., 2010); and (v) neurons containing NFT are functionally intact in cortical circuits *in vivo* (Kuchibhotla et al., 2014).

**Figure 3:**
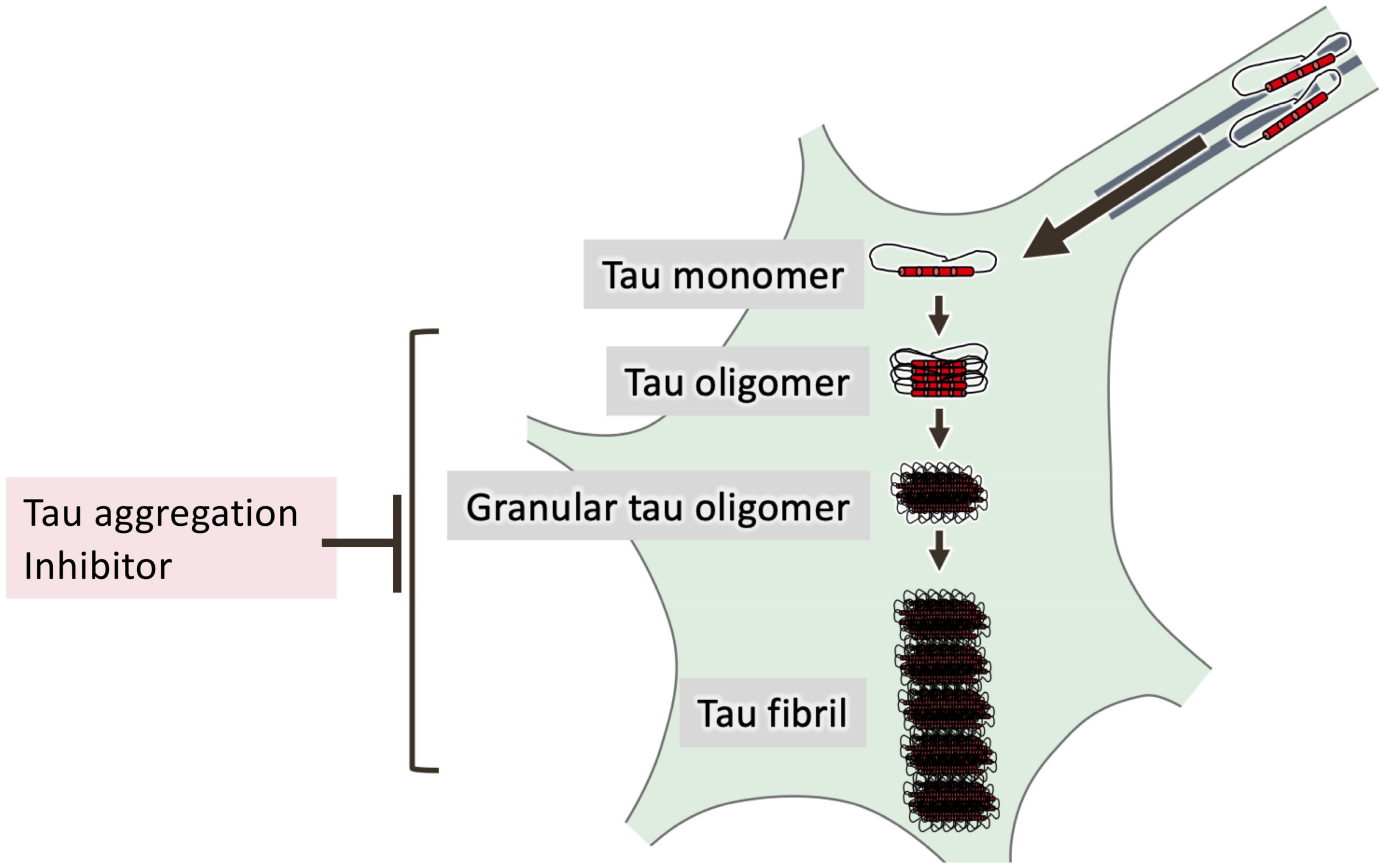
Processes of tau aggregation. Hyperphosphorylated tau is detached from microtubules and mislocalized in the somatodendritic compartment of neurons. *In vitro* studies have shown that tau is self-assembled to form tau oligomers and granular tau oligomers before forming NFTs. Tau aggregation inhibitors that halt these processes may be useful in the treatment of tauopathies.

Analysis using recombinant protein showed the existence of two different intermediate aggregates called tau oligomers and granular tau oligomers before the formation of tau filaments (Maeda et al., 2007). Importantly, granular tau oligomers can be detected in the brain before the onset of clinical symptoms of AD (Maeda et al., 2006). Analysis of wild-type tau (Kimura et al., 2007) and P301L tau transgenic mice (Kimura et al., 2010) indicated that hyperphosphorylated tau/oligomeric tau and granular tau oligomer are involved in synaptic loss and neuronal loss, respectively (Takashima, 2013). Screening of a library of natural compound derivatives identified low-molecular-weight compounds bearing a 1,2-dihydroxybenzene backbone as inhibitors of tau aggregation, specifically, of tau oligomer formation. In P301L tau transgenic mice, one such compound, DL-isoproterenol, decreased detergent-insoluble aggregated tau and neuronal cell loss (Soeda et al., 2015). Together with the more recent finding that MB reduces the number of tau fibrils and increased the number of granular tau oligomers when applied to recombinant tau protein (Soeda et al., 2019), these results support the notion that tau-mediated toxicity is not due to tau fibril formation but rather to formation of intermediate tau aggregates (Figure 3).

Immunotherapy is one potential therapeutic approach for preventing tau aggregation, a line followed by a number of academic and industrial research groups. These efforts have resulted in the generation of the TOC1 (Patterson et al., 2011), TOMA (Castillo-Carranza et al., 2014), and T22 (Lasagna-Reeves et al., 2012) antibodies that recognize intermediate tau aggregates. While ongoing tau immunotherapy-based clinical trials mainly targeted phosphorylated tau, monomeric tau, and aberrant conformational changes in tau (details below), it is likely that the next generation of antibodies will be directed at tau intermediate aggregates.

## Stabilization of Microtubules

Because axonopathy, including microtubule instability and disruption, have been observed in cultured cell models (Arawaka et al., 1999) and animal models (Spittaels et al., 1999; Probst et al., 2000) of tauopathy, and in the brains of tauopathy patients (Kneynsberg et al., 2017), microtubule stabilizers have been developed to prevent axonal/dendritic degeneration and therefore, to improve symptoms of tauopathies, including AD (Lee et al., 1994; Khanna et al., 2016). Davunetide (NAP, AL-108), an 8-amino acid peptide (Asn-Ala-Pro-Val-Ser-Ile-Pro-Gln) derived from the activity-dependent neuroprotective protein (Gozes et al., 1999), was shown to reduce tau pathology (AT8, AT180, and CP13 site-positive phosphorylated tau) and enhance cognitive function in triple-transgenic AD mice (Matsuoka et al., 2008). In a phase 2 trial of 144 patients with mild cognitive impairment (MCI), davunetide treatment for 12 weeks was shown to be safe and tolerable (Morimoto et al., 2013). However, the drug was found to be inefficacious (primary and secondary outcomes) in a phase 2/3 trial on 360 patients with PSP treated for 52 weeks (Boxer et al., 2014) and its development was discontinued.

Epothilone D (BMS-241027) is a microtubule stabilizer isolated from the myxobacterium *Sorangium cellulosum*. Epothilone D can cross the BBB (VandeVrede et al., 2020a) whereas taxanes with a similar structure are less penetrant (Fellner et al., 2002). Administration of Epothilone D reduced the number of dystrophic axons and inhibited cognitive deficits in P301S-tau transgenic mice (Brunden et al., 2010). The drug caused only one grade 3 hypersensitivity reaction when given to healthy women in a phase 1 study (VandeVrede et al., 2020a) but its development was halted without a report of its effects in a phase 1/2 study (NCT01492374; 40 patients with mild AD; Medina, 2018).

The traxane-derivative TPI287 (abeo-taxane) which has high BBB permeability proved safe and well-tolerated in a phase 1 study (NCT02133846) in patients with CBD or PSP (*n* = 44) and a phase 1 study (NCT01966666) in patients with mild to moderate AD (*n* = 33). However, the drug caused adverse effects in the AD group and worsened dementia symptoms in the CBD and PSP patients. It appears that TPI287 is no longer being developed for clinical use (VandeVrede et al., 2020a).

Recent reviews propose that tau is not a stabilizer of microtubules in the axon but rather confers flexibility to the labile domain of microtubules and leads to microtubule elongation (Qiang et al., 2018; Baas and Qiang, 2019). Further, an analysis of fast single-molecule tracking showed that microtubule assembly is regulated by more rapid tau dynamics, kiss-and-hop mechanism, than previously reported (Janning et al., 2014). These observations suggest that microtubule stabilizers may not be suitable as inhibitors of tau-related dysfunction.

## Tau Clearance and Immunotherapy

Tau is degraded in both the ubiquitin–proteasome system and the autophagy–lysosome system (Lee et al., 2013), both of which are disrupted in AD and followed by the emergence of aberrant forms of tau (Chesser et al., 2013). Previous research reported that direct or indirect enhancement of the ubiquitin–proteasome system (Shimura et al., 2004b; Myeku et al., 2016) or the autophagy–lysosome system (Majumder et al., 2011; Di Meco et al., 2017; Lauretti et al., 2017) can significantly enhance the clearance of toxic forms of tau with improvements in neuronal health and synaptic function. To date, however, drugs targeting these systems have not been tested in clinical trials. Protein clearance is enhanced by treatment with vaccines or antibodies and both, active (Troquier et al., 2012; Ando et al., 2014) and passive immunotherapies (Courade et al., 2018; Albert et al., 2019) to target tau have yielded promising results, with several clinical trials for AD and related tauopathies now in progress (Table 1) (Sandusky-Beltran and Sigurdsson, 2020).

**Table 1:** Summary of tau immunotherapies.

**Drug**	**Epitope**	**Preclinical study**	**Clinical Trial**				
			**Subject**	**Current stage**	**Trials No**.	**Sponsor/Company**	**References**
AADvac-1	Tau a.a. 294–305	AADvac-1 reduced AD-type hyperphosphorylation of tau and improved the sensorimotor functions of transgenic rats (Kontsekova et al., 2014).	AD	Phase 2	NCT02579252	Axon Neuroscience SE	Novak et al., 2017, 2018b, 2019
ACI-35	Phospho-S396/404	ACI-35 reduced insoluble tau level and improved survival in P301L tau transgenic mice (Theunis et al., 2013).	Early AD	Phase 1b/2a	NCT04445831	AC Immune SA—Janssen	
RG7345	Phospho-S422	RG7345 inhibited tau pathology in 3xTg-AD mice (Collin et al., 2014).	Healthy volunteers	Phase 1 - discontinued	NCT02281786	F. Hoffmann-La Roche	
BIIB092	Secreted N-terminal tau fragments (Tau a.a. 15–24)	IPN002, the murine analog of BIIB092, reduced the secretion of extracellular tau in cell culture and in P301L tau JNPL transgenic mice (Bright et al., 2015).	Early AD	Phase 2	NCT03352557	Biogen (Bristol-Meyers Squibb; iPerian)	Qureshi et al., 2018; Boxer et al., 2019
C2N-8E12	Extracellular form of pathological tau (Tau a.a. 25–30)	HJ8.5, the original mouse antibody of C2N-8E12, reduced tau seeding activity *in vitro* and *in vivo* (Yanamandra et al., 2013, 2015).	Early AD	Phase 2	NCT02880956 NCT03712787	AbbVie	
UCB0107	Mid-region of tau (Tau a.a. 235–246)	Antibody D, the original mouse antibody of UCB010, inhibited tau propagation *in vivo* and *in vitro* (Courade et al., 2018; Albert et al., 2019).	PSP	Phase 1	NCT04185415	UCB Biopharma	
LY3303560	Same as MC1 antibody (Tau a.a. 7–9, 313–322)	MC1 injection reduced tau pathology in tau transgenic mice (Chai et al., 2011; d'Abramo et al., 2013).	Patients with early symptomatic AD	Phase 2	NCT03518073	Eli Lilly	
BIIB076	Monomeric and fibrillar tau		Healthy volunteers and AD	Phase 1	NCT03056729	Biogen	
JNJ-63733657	Mid-region of tau		Healthy subjects and AD	Phase 1	NCT03375697 NCT03689153	Janssen	
Lu AF87908	Phospho-S396	The original mouse antibody inhibited tau propagation *in vitro* and *in vivo* (Rosenqvist et al., 2018).	Healthy subjects and AD	Phase 1	NCT04149860	H. Lundbeck A/S	
PNT001	*cis*-phospho-T231	The original mouse antibody improved traumatic brain injury-related structural and functional sequelae in a mouse model (Kondo et al., 2015).	Healthy volunteers	Phase 1	NCT04096287	Pinteon Therapeutics	
RO7105705	N-terminal region of tau	RO7105705 reduced brain pathology in P301L tau transgenic mice (Lee et al., 2016).	AD	Phase 2	NCT03289143 NCT03828747	AC Immune SA—Genentech—F. Hoffmann-La Roche	Kerchner et al., 2017

*3xTg-AD mice harbor a PSEN1 mutation (M146V) and the co-injected Swedish mutant amyloid precursor protein and tauP301L transgenes*.

### Active Immunotherapy (Vaccinations)

#### AADvac-1

Active immunization is an attractive therapeutic approach because it can induce a sustained autoantibody response in small doses. Moreover, unlike passive immunity, the therapeutic effects should not be limited by the production of anti-drug antibodies.

The first approach for tau active immunization was to immunize normal C57BL6J mice with full-length recombinant human tau whose response included the display of encephalomyelitis, axonal damage, and inflammation (Rosenmann et al., 2006). To circumvent these effects, active immunization subsequently used fragmented tau or phosphorylated peptides of tau. Injection of a 30-amino acid tau phospho-peptide (aa 379–408 residues, including phospho-S396 and S404) into P301L tau transgenic mice (JNPL3) reduced aggregated tau in the brain and slowed progression of the tangle-related behavioral phenotype (Asuni et al., 2007). AADvac1, a synthetic peptide consisting of amino acids 294–305 of the tau (KDNIKHVPGGS) reduced tau pathology and pathology-associated behavioral deficits in transgenic rats (Kontsekova et al., 2014). The vaccine was the first anti-tau vaccine to enter clinical trials in 30 patients with mild to moderate AD, and had a favorable safety profile and excellent immunogenicity. A phase I pilot trial of AADvac1 (40 or 160 μg) for 2 years in patients (*n* = 30) with the non-fluent/agrammatic variant of primary progressive aphasia was conducted (AIDA) (NCT03174886). A phase 2 trial to evaluate its safety and efficacy was conducted in patients with mild AD for 24 months (ADAMANT) (NCT02579252). Axon Neuroscience presented, at the 2020 virtual AAT-AD/PD Focus Meeting, that the incidence of adverse events under the treatment of the antibody did not differ from the placebo group. More than 80% of participants immunized by the vaccine acquired high-affinity tau antibodies. In the trial, AADvac-1 decreased the level of blood neurofilament, a marker for neurodegeneration. While the vaccine treatment did not improve the cognitive score in an analysis for participants of all ages, a preplanned age subgroup analysis showed that treatment trended to slow cognitive decline among younger participants. The vaccine treatment decreased phosphorylated tau levels in cerebrospinal fluid (CSF), but the CSF changes were not statistically significant. Axon Neuroscience is appeared to plan a phase 3 trial (Alzforum.org, Therapeutics; AADvac1. https://www.alzforum.org/therapeutics/aadvac1).

#### ACI-35

ACI-35 is a liposomal vaccine developed by AC Immune (Switzerland); its constituents include tau fragment (393–408) with several phosphorylated serine residues (S396/S404). In December 2013, AC Immune initiated a 6 month phase 1b study to compare low-, moderate-, and high-dose ACI-35 in mild to moderate AD subjects (ISRCTN, ISRCTN13033912) (Alzforum.org, Therapeutics; ACI-35. https://www.alzforum.org/therapeutics/aci-35). Results, presented at the virtual AAT-AD/PD Focus Meeting in 2020, showed that although the vaccine was well-tolerated, it elicited a weak immune response (Alzforum.org, news; https://www.alzforum.org/news/conference-coverage/active-tau-vaccine-hints-slowing-neurodegeneration). ACI-35 was redesigned, and ACI-35.030 was produced. This showed a more robust immune response than ACI-35 in rhesus monkeys. The vaccine is now licensed to Janssen, and a multicenter phase 1b/2a study is being conducted to evaluate the safety and immunogenicity of this vaccine in AD patients (NCT04445831). In July 2020, AC Immune firstly announced that positive safety and immunogenicity data were obtained in the lowest dose of ACI-35.030 (AC immune press release; https://ir.acimmune.com/news-releases/news-release-details/ac-immune-advances-phospho-tau-alzheimers-vaccine-phase-1b2a).

#### Passive Immunotherapy

The target for passive immunotherapy is commonly an extracellular protein because antibodies having a molecular weight of ~150 kDa cannot efficiently penetrate cells. Studies in AD patients (Braak and Braak, 1995) as well as *in vitro* (Frost et al., 2009) and *in vivo* (Clavaguera et al., 2009), have shown that the spread of tau accumulation in tauopathy is related to the prion-like properties of tau (Mudher et al., 2017; Duyckaerts et al., 2019). Thus, the tau propagation hypothesis posits that pathological forms of tau (tau seeds) released from donor cells can be taken up by recipient cells where they induce the formation of intracellular tau aggregates. Based on this, tau passive immunotherapy is considered a suitable treatment for removing extracellular tau (Colin et al., 2020). On the other hand, anti-tau monoclonal antibodies reportedly invade neurons, probably through clathrin-mediated endocytosis, indicating that intracellular tau can also be targeted by tau antibodies (Congdon et al., 2013). At present, there are 10 tau antibodies that have entered clinical trials.

##### RG7345 (RO6926496)

The first passive immunization test in a clinical trial (RO6926496) was carried out with the antibody RG7345 which targets phosphorylated tau at the S422 residue found in pathological tau aggregates. In a triple-transgenic mouse model harboring three mutations (presenilin 1 M146V, Swedish mutant amyloid precursor protein (APP), and tauP301L), RG7345 was previously shown to inhibit tau pathology (Collin et al., 2014). A phase 1 trial by Hoffman-La Roche in healthy volunteers was initiated to evaluate the safety, tolerability, and pharmacokinetics of RG7345 (NCT02281786). The development of this drug has been discontinued. While the results have not been published to the best of our knowledge, negative speculations about the antibody's pharmacokinetic has been expressed (Congdon and Sigurdsson, 2018; Sandusky-Beltran and Sigurdsson, 2020).

##### BIIB092 (BMS-986168, IPN007, Gosuranemab)

BIIB092 is a humanized monoclonal antibody against an N-terminal fragment of tau (extracellular tau) secreted from familial AD patient-derived pluripotent stem cells. The epitope is considered to be N-terminal amino acids 15–24 and notably, IPN002, the murine analog of BIIB092 was reported to reduce the secretion of extracellular tau in cell culture and in P301L tau JNPL transgenic mice (Bright et al., 2015). Results from phase 1 trials (NCT02294851) (NCT02460094) indicate that BIIB092 is safe and well-tolerated (Qureshi et al., 2018; Boxer et al., 2019). The phase 1 trial reported a marked reduction in CSF-free N-terminal tau post-immunization in healthy participants (Qureshi et al., 2018) and PSP (Boxer et al., 2019). The PASSPORT (NCT03068468) phase 2 trial, conducted in PSP patients was discontinued however because of a lack of efficacy in the interim analysis (Sandusky-Beltran and Sigurdsson, 2020). The lack of efficacy might be explained by the fact that the amount of CSF tau did not change between the control subjects and PSP patients (Sandusky-Beltran and Sigurdsson, 2020) and that IPN002 did not reduce amounts of intracellular free tau in cultured cells (Bright et al., 2015). BIIB092 is in a phase 2 clinical trial for AD (TANGO; NCT03352557).

##### C2N-8E12 (ABBV-8E12)

C2N-8E12 is a humanized immunoglobulin (Ig)G4 antibody that recognizes an aggregated extracellular form of pathological tau. HJ8.5, the original mouse antibody of C2N-8E12, reduced tau seeding activity *in vitro* (Yanamandra et al., 2013) and *in vivo* (Yanamandra et al., 2013, 2015). Epitope of HJ8.5 was at residues 25–30 aa (Yanamandra et al., 2013). While a phase 1 trial (NCT03413319) in PSP patients showed safety and good tolerability (Sandusky-Beltran and Sigurdsson, 2020), interim results of a phase 2 trial (NCT02985879) in patients with PSP symptoms for <5 years failed to find any therapeutic beneficial effects of this antibody (Alzforum.org, News, https://www.alzforum.org/news/research-news/abbvies-tau-antibody-flops-progressive-supranuclear-palsy), leading to a discontinuation of the PSP trial (Sandusky-Beltran and Sigurdsson, 2020). However, trials with C2N-8E12 in AD patients were conducted (NCT02880956). An extension study (NCT03712787) is being conducted for patients who have successfully completed the phase 2 trial to evaluate long-term safety and tolerability.

##### UCB0107

The monoclonal antibody UCB0107 binds to the mid-region of tau (amino acids 235–246). Its preceding mouse version (antibody D) reportedly inhibited transneuronal propagation of pathogenic and aggregated tau *in vivo* (Albert et al., 2019), and seeding activity of human AD and PSP tau *in vitro* (Courade et al., 2018). Two phase 1 studies aimed to evaluate the safety, tolerability, and pharmacokinetic properties of UCB0197 in healthy adult males were conducted (NCT03464227 and NCT03605082). A phase 1 study is ongoing in patients with PSP (NCT04185415). The results of the trials are not yet available.

##### LY3303560 (Zagotenemab)

LY3303560 is a humanized anti-tau monoclonal antibody, its prototype being monoclonal anti-mouse MC1 which recognizes conformation-specific epitopes that appear along with tau aggregation. *In vivo* experiments showed that MC1 injection reduced tau pathology in tau transgenic mice (Chai et al., 2011; d'Abramo et al., 2013) and since MC1 antibodies are not incorporated by neurons (d'Abramo et al., 2013), their mechanism of action is thought to involve binding and removal of extracellular PHF tau. The epitopes recognized by LY3303560 are two discontinuous portions of tau, 7–9 and 313–322 amino acid residues (Jicha et al., 1997, 1999). LY3303560 binds preferentially to tau aggregates rather than monomers. To date, a phase 1 trial of LY3303560 has been conducted to evaluate safety in MCI and mild to moderate AD (NCT03019536) while an ongoing phase 2 trial (NCT03518073) is being undertaken in early symptomatic AD patients. The results of both studies are currently awaited.

##### BIIB076

BIIB076 is a human recombinant monoclonal anti-tau antibody. Although the epitope to which this antibody is directed has not been revealed, it is known that it recognizes monomeric and fibrillar tau (Alzforum.org, Therapeutics; BIIB076. https://www.alzforum.org/therapeutics/biib076). So far, a phase 1 trial to examine the safety of BIIB076 has been conducted in healthy volunteers and AD patients (NCT03056729).

##### JNJ-63733657

JNJ-63733657 is a monoclonal antibody that recognizes the central domain of tau (Sigurdsson, 2018), but the exact epitope is unknown and results of preclinical testing are not available. A phase 1 trial of JNJ-63733657 in healthy volunteers and patients with prodromal or mild AD has been conducted (NCT03375697 and NCT03689153).

##### Lu AF87908

The monoclonal antibody Lu AF87908 binds to the phospho-S396 region of tau (Sandusky-Beltran and Sigurdsson, 2020). A preclinical study showed that the original mouse antibody inhibited tau propagation induced by exposure of tau transgenic brain lysates *in vivo* and *in vitro* (Rosenqvist et al., 2018). Currently, a phase 1 trial (NCT04149860) is assessing the tolerability of Lu AF87908 in healthy individuals and AD patients (NCT04149860).

##### PNT001

Phosphorylated T231 on tau has a cis- or trans-structure (Albayram et al., 2016), but only the cis form appears early in the brains of patients with MCI where it is localized to dystrophic neurites during the progression of AD (Nakamura et al., 2012). Monoclonal antibodies against cis-phospho-T231 improved traumatic brain injury-related structural and functional sequelae in a mouse model (Kondo et al., 2015) of chronic traumatic encephalopathy (CTE) in which tau pathology is observed (Katsumoto et al., 2019). Currently a phase 1 study is being undertaken in healthy volunteers to evaluate the safety, tolerability, and pharmacokinetics of this antibody (NCT04096287).

##### RO7105705 (MTAU9937A, RG6100, Semorinemab)

RO7105705 is a monoclonal IgG4 antibody, which is modified to reduce effector function, such as microglial activation (Lee et al., 2016). This antibody recognizes N-terminus on tau and reacts with all six isoforms of human tau, both with or without phosphorylation. Further, the antibody can bind monomeric and oligomeric tau, and reduced brain pathology in P301L tau transgenic mice (Alzforum.org, THERAPEUTICS, https://www.alzforum.org/therapeutics/semorinemab). A phase 1 study was conducted in healthy volunteers and patients with mild-to-moderate AD (Kerchner et al., 2017) (NCT02820896). The trial showed that no serious adverse events occurred, and RO7105705 plasma half-life was 32 days. Two phase 2 trials were conducted in AD patients to evaluate efficacy and safety (NCT03289143 and NCT03828747). Genentech announced top-line results from a phase 2 trial. Unfortunately, RO7105705 did not meet the primary efficacy end-point of reducing the decline of the cognitive score (Genentech press release).

Tau immunotherapy can directly target tau via binding to specific sequences or specific conformational changes. Since phosphorylation of S396/S404 (Augustinack et al., 2002) and S422 (Augustinack et al., 2002; Vana et al., 2011; Kanaan et al., 2016) was increased with the progression of AD (Augustinack et al., 2002; Vana et al., 2011) and CTE (Kanaan et al., 2016) stage, antibodies such as Lu AF87908 and ACI-35 target phosphorylation may be effective in halt progression of the disease. Antibodies that bind to the N-terminus region or mid-domain region target extracellular tau. Since an antibody against the mid-region of tau (antibody D) was more efficient at preventing both seeding and propagation (Albert et al., 2019) than were antibodies targeting the N-terminal region or C-terminal region (Nobuhara et al., 2017; Courade et al., 2018), mid-domain targeted antibodies, such as JNJ-63733657 and UCB0107 may be more effective than N-terminal-directed antibodies at disrupting the seeding and propagation of aberrant tau. Recently, Genentech showed no efficacy of the N-terminal-directed antibody, RO7105705, in a phase 2 trial.

## Reducing Tau Expression by Oligonucleotide Therapy

Oligonucleotide therapy, including antisense and Si-RNA, is a new strategy for difficult-to-treat hereditary diseases. The therapies aim to control the onset and progression of the disease by regulating protein expression levels. Previous reports using tau-knockout mice have shown a beneficial effect on the electrophysiological and/or behavioral deficits in models of AD (Roberson et al., 2007; Yoshikawa et al., 2018). In P301S tau transgenic mice, tau antisense oligonucleotides (ASO) reduced the amounts of tau mRNA by ~50%, inhibiting hippocampal atrophy, neuronal loss, and a behavioral abnormality (DeVos et al., 2017). Immunocytochemical analysis revealed that stereotactic injections of Si-RNA against tau into the brains of the mice suppress pathological tau phosphorylation (AT180 and CP13) and conformational changes in tau (MC1), suggesting that potential gene therapeutic value of Si-RNA against tauopathies (Xu et al., 2014). The ASO drug BIIB080 is in a phase 1/2, double-blind, placebo-controlled trial (NCT03186989) (Alzforum.org, THERAPEUTICS, https://www.alzforum.org/therapeutics/biib080; https://ir.ionispharma.com/static-files/4ab8c591-c51b-45e1-8b0d-ef83a46c0853) in which subjects with mild AD (aged 50–74 years old) are being intrathecally injected with BIIB080 to evaluate safety and pharmacokinetics.

Regulation of tau alternative splicing may also be worthy of exploration for the treatment of tauopathies. In FTDP-17, an inherited tauopathy, single nucleotide polymorphisms [SNP, ≥47 (Rossi and Tagliavini, 2015)] are present on the *MAPT* gene are causally related to a wide variety of clinical symptoms. Many SNPs are located on exon 10 and intron 10. Some of these SNPs (N279K, N296H, ΔK280) alter the ratio of 3R and 4R tau isoforms (Andreadis, 2012): whereas the N279K or N296H mutations increased 4R tau transcripts, a ΔK280 decreased the number of 4R tau transcripts (Rossi and Tagliavini, 2015). The ratio of tau isoforms is also altered in other primary tauopathies. For example, while PSP and CBD predominantly exhibit 4R tau, the insoluble tau in the brain of PiD is mainly 3R tau (Arai et al., 2003). Therefore, oligonucleotide therapy, which can specifically reduce 4R or 3R tau, is useful in treating tauopathies. In a mouse model of human tau expression, ASO treatment significantly shifted the splicing pattern to lower 4R tau without altering the abundance of total tau (Schoch et al., 2016) and led to a phenotypic improvement (Schoch et al., 2016). Importantly, because of the physiological functions of tau proteins, such as synaptic plasticity (Kimura et al., 2014b), signaling (Marciniak et al., 2017) and nucleic acid protection (Sultan et al., 2011; Violet et al., 2014), close monitoring of adverse effects of oligonucleotide therapies is essential.

## New Tau-Target Therapy for Tauopathies

### Liquid–Liquid Phase Separation (LLPS)

In addition to the above, candidate therapeutic targets for tauopathies include tau truncation (Novak et al., 2018a), impairment of axonal transport (Combs et al., 2019), dysfunction of tau in the nucleus (Bukar Maina et al., 2016), and functional impairment of dendritic tau (Ittner and Ittner, 2018). As previously mentioned, tau protein is a large intrinsically disordered protein (IDP) (Tompa, 2002) that can interact with multiple binding partners (Morris et al., 2011; Guo et al., 2017; Salvi, 2019). Tau is well-known to partner with microtubules (Butner and Kirschner, 1991; Gustke et al., 1994) and also interacts with other cytoskeletal proteins, such as actin (Griffith and Pollard, 1978; Fulga et al., 2007; Whiteman et al., 2009), and neurofilaments (Mandelkow and Mandelkow, 2012). Tau also interacts with kinases involved in post-translational modification of tau (Takashima et al., 1998; Sun et al., 2002), chaperones (Shimura et al., 2004a; Dickey et al., 2007) and nucleic acids (Loomis et al., 1990; Kampers et al., 1996; Sultan et al., 2011).

Currently, many cell biologists are turning their attention to liquid–liquid phase separation (LLPS) which can make a non-membrane-bound compartment in cells via liquid–liquid de-mixing and separation from the liquid cytoplasm (Hyman et al., 2014). Change of phase transition state by LLPS leads to formation of a protein condensate referred to as liquid droplets. These droplets are transient in nature owing to their weak interactions (electrostatic, cation-π, and π-π) (Brangwynne et al., 2015). In 2015, Uversky et al. hypothesized that intrinsically disordered IDP serve as essential drivers of intracellular LLPS that generate various membrane-less organelles (Uversky et al., 2015). Abnormal LLPS is associated with neurodegenerative diseases; for example, RNA-binding protein fused in sarcoma has a low-complexity domain within the N-terminal domain, which is SYGQ-rich (serine-, tyrosine-, glycine-, glutamine-rich), can be transformed to liquid droplets through LLPS (Patel et al., 2015) and converted to an aggregate state over time as seen in amyotrophic lateral sclerosis patients (Patel et al., 2015). Other IDP, including TAR DNA-binding protein-43 (Mann et al., 2019) and heterogeneous nuclear ribonucleoprotein A1 (Molliex et al., 2015) with a low-complexity domain, have also been reported to form liquid droplets by LLPS and are involved in forming pathological inclusions. These findings suggest that LLPS is an essential physiological and pathological event and indeed, several studies have shown that LLPS-mediated conversion of tau protein to liquid droplets (Ambadipudi et al., 2017; Zhang et al., 2017; Wegmann et al., 2018; Boyko et al., 2019; Kanaan et al., 2020).

Stress granules are membrane-less organelles produced via LLPS. In stress granules, proteins and RNA interact with many partners to form reversible complexes. The granules bear a cytoprotective function against stress since they temporarily inhibit the translation of non-essential mRNA and promote translation of transcripts necessary for cell survival (Kedersha et al., 2000; Liu-Yesucevitz et al., 2011). Tau has been shown to undergo polymerization by RNA (as well as heparin) (Kampers et al., 1996; Wang et al., 2006). Tau can binds to RNA in living cells (Zhang et al., 2017) and it has been shown that multiple tau molecules bound to tRNA leads to an increase in tau levels and formation of coacervates (Kosik and Han, 2019); thus, incubation of a tau-poly (U) RNA complex under droplet-forming conditions results in a gradual increase in thioflavin T (ThT) fluorescence over 15 h. These observations have been supported by others showing that the formation of seeding-competent tau aggregates from tau condensates (Wegmann et al., 2018). Thus, the fact that β-sheets form by prolonged retention of tau droplets, suggests that the highly condensed-phase state of tau is a precursor to protofibrillation. Tau LLPS may be initiated by localized gathering of tau into cellular compartments (e.g., dendrites). Although the physiological intracellular concentration of tau has been suggested to be ~2 μM (Wegmann et al., 2018), more than 50% of tau molecules are bound to microtubules (Ackmann et al., 2000). During NFT formation, non-fibrillar and hyperphosphorylated tau accumulate in the soma and dendrites of neurons (Götz et al., 1995; Uchihara et al., 2001). Therefore, tau dissociated from microtubules migrates into the cytoplasm and forms the first aggregates. Presumably, the localized gathering of tau is a singularity of aggregation, but the mechanism is unknown.

Molecular crowding tunes the physical properties of RNP condensates (Kaur et al., 2019). Different independently-performed studies have reported the formation of tau liquid droplets *in vitro* (tau concentrations from 1 to 25 μM) in the presence of crowding agents that mimic molecular crowding (~100–200 mg/ml proteins) in an intracellular environment (Hernández-Vega et al., 2017; Wegmann et al., 2018). Tau phosphorylation by microtubule affinity-regulating kinase 2, which dissociates tau from microtubules, is converted to liquid droplets (Wegmann et al., 2018). Together, these findings suggest that LLPS is involved in local tau localization (Wegmann, 2019).

**Figure 4:**
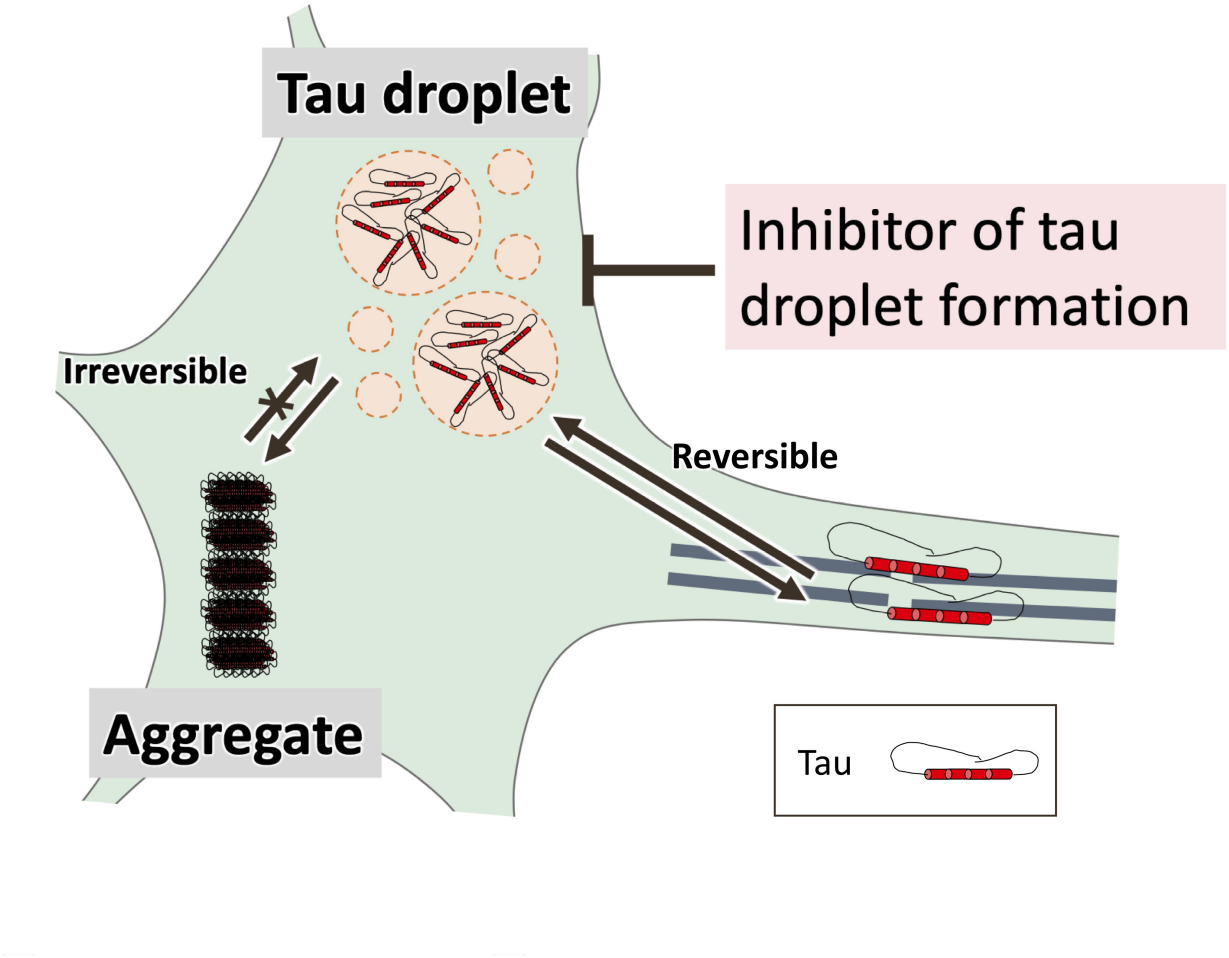
Tau droplet formation by liquid-liquid phase separation (LLPS) is a key initial step in aberrant tau aggregation. Tau must be abundant before it begins to aggregate. LLPS-mediated formation of droplets supersaturated with tau may be a key step in the latter process. A droplet is a reversible structure formed by weak interactions, suggesting that tau droplets may be a better drug target than irreversible tau aggregation.

A relationship between tau and stress granules has been reported (Apicco et al., 2018; Piatnitskaia et al., 2019) in which T-cell intracellular antigen-1 (TIA-1), a DNA/RNA-binding protein transferred from the nucleus to the cytoplasm during stress may play an important role (Kedersha et al., 2000). In fact, TIA-1 co-localizes with pathological tau in human tissue from AD patients (Vanderweyde et al., 2012), and a 50% reduction in TIA-1 expression of cytoplasmic stress granules in P301S tau transgenic (PS-19) mice leads to improvements in behavior and lifespan in parallel with reduced neuronal and synaptic degeneration (Apicco et al., 2018). In cultured hippocampal neurons, knockdown or knockout of TIA-1 also reduced tau misfolding and toxicity, and kinase inhibitors that reduce stress granule formation reduced tau abnormalities (Vanderweyde et al., 2016). Therefore, inhibition of tau droplet formation could be an important focus of therapeutic developments for tauopathies. Since a droplet is a reversible structure formed by weak interactions, tau droplets may be a better drug target than irreversible tau aggregation (Figure 4). On the other hand, since it has been shown that the condensed tau phase is involved in the nucleation of microtubules *in vitro* (Hernández-Vega et al., 2017), identification of the droplets formed specifically in tauopathies and the search for inhibitors of the droplets will be a challenge for the future.

### Inflammation

GWAS studies reported 40 risk loci associated with AD (Andrews et al., 2020), many of which appear to be associated with immune response/inflammation, APP processing, endocytosis, lipid metabolism, tau pathology, and cell migration (Ikezu and Gendelman, 2016). Several reports link dysregulation of inflammation with AD (McGeer et al., 1989; Akiyama et al., 2000; Kinney et al., 2018). In the central nervous system, microglia have major roles in the inflammatory process (Kim and Joh, 2006). Carriers of the R47H allele on TREM2 have a 2 to 4.5-fold increased risk of developing late-onset AD, suggesting that TREM2 is the second most relevant risk gene after apolipoprotein E-ε4 (Kinney et al., 2018). TREM2 is specifically expressed in microglia and is essential for maintaining microglial metabolic fitness during stress events (Ulland and Colonna, 2018). In mice expressing humanized TREM2, R47H impairs TREM2 function, restores Aβ-induced microgliosis and microglial activation (Song et al., 2018). Some authors, studying the role of TREM2 in tauopathies, have reported that TREM2 deficiency exacerbates tau pathology [e.g., tau hyperphosphorylation and aggregation via activated neuronal stress kinases (Bemiller et al., 2017)]. Further, TREM2 deficiency was reported to attenuate neuroinflammation and protects against neurodegeneration in P301S mutant human tau transgenic (PS19) mice (Leyns et al., 2017). Even though the role of TREM2 in tau pathology and tau-mediated toxicity remains a matter of debate, it is notable that a correlation between microglia activation and formation of pathological tau has been recorded (Laurent et al., 2018). Other recent studies have focused on the link between NLRP3 inflammasomes formed within the microglia and tau. Extracellular tau monomers and oligomers stimulate NLRP3 activation, increasing tau hyperphosphorylation. Since loss of function of the NLRP3 inflammasome has been shown to reduce tau pathology (Ising et al., 2019; Zhang et al., 2020b), it appears that properly-regulated inflammation may protect against tau pathology and tau-mediated toxicity.

### Tau Loss of Function

A number of studies have suggested that correcting the loss of tau function may be valuable or more valuable than correcting the toxic gain-of-tau function (including hyperphosphorylation and aggregation), in drug discovery programs for tauopathies (Trojanowski and Lee, 2005). The majority of tau-targeted drugs are designed to reduce the gain-of-toxic-tau and increase the amount of normal tau (e.g., microtubule stabilizers). Since the 2010's, a number of studies have pointed to the role of tau in dendrites and nuclei. For example, *in vivo* and *ex vivo* electrophysiological studies have shown dysregulation of synaptic plasticity to be associated with tau deficiency (Ahmed et al., 2014; Kimura et al., 2014b; Regan et al., 2015; Marciniak et al., 2017) and studies in a tau deficiency model showed that nuclear tau is protective against the cellular response to stress (Sultan et al., 2011; Violet et al., 2014). These findings indicate that tau has other physiological functions other than its better-known microtubule-stabilizing effects; therefore, treatments that restore tau function may be helpful the management of tauopathies.

## Discussion

There has been great interest in conducting tau-targeted therapeutics in clinical trials to slow the onset and progression of tauopathies such as AD (Giacobini and Gold, 2013). These have been driven by the fact that phase 3 clinical trials for AD using Aβ-targeted drugs failed to reach their primary predicted outcome (Holmes et al., 2008; Rosenblum, 2014). Importantly, the shift in focus on tau has led to the discovery of atypical tau functions (Sotiropoulos et al., 2017) that may uncover new strategies for preventing and treating AD.

Recent electron cryomicroscopy (cryo-EM) studies on the structure of tau fibrils in postmortem brain samples from tauopathy patients have provided interesting perspectives into the direction of current and future tau-targeted therapies. The pattern of tau isoform deposition varies with each tauopathy; all six isoforms are presented in AD and CTE, and 3R-tau and 4R-tau are predominately expressed in Pick's disease and CBD, respectively. In 3R/4R tauopathies, the tau filament folds are very similar to those found in AD (Fitzpatrick et al., 2017) and CTE (Falcon et al., 2019), although the filament folds in Pick's disease (Falcon et al., 2018) and CBD (Zhang et al., 2020a) differ from those observed in 3/4R tauopathies and the tau filament core in Pick's disease is distinct from that in CBD. These observations suggest that different tau sequences must be targeted in 3/4R, 3R, and 4R tauopathies. A combination of cryo-EM and mass spectrometric post-translational modification mapping indicate that ubiquitination of tau can mediate inter-protofilament interfaces in fibrils from CBD and AD (Arakhamia et al., 2020). While classical biochemical assays have detected tau ubiquitinations in PHF from AD (Mori et al., 1987; Morishima-Kawashima et al., 1993), a re-focusing on the regulation of tau ubiquitination may help efforts to inhibit tau aggregation.

The ability of immunotherapies (vaccines and antibodies) to reach the brain at sufficient doses remains questionable. Preclinical studies have shown that CSF penetration of some IgG monoclonal antibodies is only 0.1–1% (Shin et al., 2020a), prompting efforts to improve BBB permeability by designing bi-specific antibodies with a therapeutic arm and a BBB-crossing arm (targeting the transferrin receptor and insulin receptor) have been developed (Neves et al., 2016). The presence of tau released from neurons in the CSF (Meredith et al., 2013; Sato et al., 2018) and interstitial fluid (Yamada et al., 2011, 2014) has led to the development of immunotherapies targeting extracellular tau. Since targetable extracellular tau is estimated to be 0.01–0.001% of intracellular tau, targeting intracellular tau has been suggested to be a more promising approach (Sandusky-Beltran and Sigurdsson, 2020). Conventional monoclonal antibodies are 150-kDa multimeric proteins containing a heavy chain and a light chain. To reduce manufacturing costs and to target previously neglected molecules, smaller recombinant antibody fragments, such as antigen-binding fragment and single-chain variable fragment (scFv), have been developed. The US FDA has approved two scFvs: brolucizumab, a humanized scFv for neovascular (wet) age-related macular degeneration, and blinatumomab, a mouse scFv for acute lymphoblastic leukemia. Camelid single-domain (12–15 kDa) antibodies (sdAbs or VHHs, also widely known as nanobodies) have full antigen-binding potential and strong affinity to their cognate antigen (Arbabi-Ghahroudi, 2017; Jovčevska and Muyldermans, 2020). Caplacizumab, a nanobody, has been approved by the EMA and FDA to treat thrombotic thrombocytopenic purpura. Although an ScFv could be used as an intrabody (an intracellular antibody), they are destabilized by intracellular reduction and often fail to function as antibodies (Stocks, 2005). Kabayama and colleagues reported a method for engineering an ultra-stable cytoplasmic antibody (STAND), which fuses scFv into peptide tags with a highly negative charge and a low isoelectric point (Kabayama et al., 2020). It has been shown that STAND-A36 binds to the presynaptic vesicle protein synaptotagmin and inhibits dopamine release in cultured cells (Kabayama et al., 2020). Since tau is an intracellular protein, gene therapy, in which cDNA expressing a tau intrabody is loaded into neurons, may also prove efficacious for treating tauopathies.

We are attempting to approach both tau gain- and loss-of-function to discover the therapeutic potential of tau. One tau gain-of-function is tau oligomer formation that emerges before forming tau fibrils. In particular, granular tau oligomers have been suggested to be correlated with neuronal loss (Takashima, 2013). We found that isoproterenol reduces granular tau oligomer formation and inhibits cell death and behavioral disorders (Soeda et al., 2015). Recently, we screened monoclonal antibodies that recognize granular tau oligomers, which may be used to develop therapeutic agents in the future. We reported that tau has a critical physiological function in LTD (Kimura et al., 2014b). We are currently investigating the mechanism, which may lead to discovering new drugs targeting tau loss-of-function.

Clinical trials of tau-based drugs aimed at gain-of-toxic-tau function (e.g., dysregulation of post-translational modifications and tau aggregation) or loss-of-function (microtubule instability) have been conducted in tauopathy patients. Once these clinical trials have been completed, the potential benefit of tau in the treatment of progressive neurodegenerative dementias may be revealed. However, even if these trials fail, they will serve as a foundation for the next generation of tau-based drugs. New tau abnormalities in the pathological and (previously unknown) physiological functions of tau have been reported in many papers.

The research reviewed here amply shows that basic research related to tau drug discovery, integration of research results, and clinical studies on candidate therapeutics must continue in our effort to treat tauopathies.

## Author Contributions

YS and AT conceived and designed this article and revised and approved the final version of the manuscript revision. YS drafted the manuscript. All authors contributed to the article and approved the submitted version.

## Conflict of Interest

The authors declare that the research was conducted in the absence of any commercial or financial relationships that could be construed as a potential conflict of interest.

## Abbreviations

a. a.: amino acid
Aβ: amyloid-β
AD: Alzheimer's disease
APP: amyloid precursor protein
ASO: antisense oligonucleotides
CBD: corticobasal degeneration
cryo-EM: electron cryomicroscopy
CSF: cerebrospinal fluid
CTE: chronic traumatic encephalopathy
EMA: European Medicines Agency
FDA: US Food and Drug Administration
FTDP-17: frontotemporal dementia and parkinsonism linked to chromosome 17
GSK3: glycogen synthase kinase 3
IDP: intrinsically disordered protein
Ig: immunoglobulin
LLPS: liquid–liquid phase separation
MB: methylene blue
MCI: mild cognitive impairment
NFT: neurofibrillary tangles
OGA: O-GlcNAcase
O-GlcNAcylation: addition of β-linked N-acetylglucosamine
PHF: paired helical filaments
PiD: Pick's disease
PMDA: Pharmaceuticals and Medical Devices Agency
PP2A: protein phosphatase 2A
PSP: patients with progressive supranuclear palsy
3R: three microtubule-binding repeats
4R: four microtubule-binding repeats
scFv: single-chain variable fragment
SF: straight filaments, STAND, ultra-stable cytoplasmic antibody.
